# Supplementary material for: Low-dose ionizing radiation generates a hormetic response to modify lipid metabolism in Chlorella sorokiniana
Source: Commun Biol. 2024 Jul 6;7:821. doi: 10.1038/s42003-024-06526-6 (PMC11226653; doi:10.1038/s42003-024-06526-6)
Supplement: Supplementary file 2 — Supplementary information [file 42003_2024_6526_MOESM2_ESM.pdf]

Supplementary information

Supplementary Table 1. Doses and dose rates of X-radiation that were applied and presented in this study.
The dose of irradiation that was absorbed by the sample was calculated by subtracting the doses measured by dosimeter in the absence (emitted doses) and the presence of the sample.

Emitted doses (Gy)	Dose rates of emitted radiation (Gy/min)	Time of irradiation (s)	Doses absorbed by the sample (Gy)	Approximate doses presented (Gy)	Dose rates of absorption of irradiation (Gy/min)	Approximate dose rates presented (Gy/min)
10	0.565	1062	1.082	1	0.061145	0.05
20	0.565	2124	2.165	2	0.061145	0.05
50	0.565	5310	5.411	5	0.061145	0.05
100	0.565	10620	10.823	10	0.061145	0.05
10	2.29	262	1.068	1	0.24458	0.25
20	2.29	542	2.209	2	0.24458	0.25
50	2.29	1310	5.340	5	0.24458	0.25
100	2.29	2620	10.680	10	0.24458	0.25
10	5.057	120	1.101	1	0.550305	0.5
20	5.057	240	2.201	2	0.550305	0.5
50	5.057	594	5.448	5	0.550305	0.5
100	5.057	1186	10.878	10	0.550305	0.5
200	5.057	2372	21.756	20	0.550305	0.5

Supplementary Table 2. Primer sequences used for qPCR.

Gene ID	Primer name	Sequence (5′ – 3′)	Tm (°C)	GC (%)	Product size (bp)
C2E21_8111	Primer_8111_F	CAGTCTGGCTGCTGATGAAG	59.7	55.0	158
	Primer_8111_R	CCCAGTGGTGAACACACAAG	60.0	55.0	
C2E21_8911	Primer_8911_F	CAAGGGCAGGTCTGTAGCTC	60.0	60.0	159
	Primer_8911_R	GACTGACCAAAGGCGAAGAC	59.9	55.0	
C2E21_4894	Primer_4894_F	CGCAGCTTCCTCATCTATCC	59.9	55.0	233
	Primer_4894_R	TCCTCAAAGCTGGGGTACTG	60.2	55.0	
C2E21_4991	Primer_4991_F	TCACCCAGGAGGAGATCAAG	60.2	55.0	167
	Primer_4991_R	CACCAGGTAGTCGGGGTAGA	60.0	60.0	
C2E21_7776	Primer_7776_F	TGAGCAACGGAGAGAATGTG	60.0	50.0	154
	Primer_7776_R	TCTTTAGCCAGCTCCTCCAG	59.7	55.0	
C2E21_5496	Primer_5496_F	CCTACAAGCTGTCCCTGCTC	60.0	60.0	166
	Primer_5496_R	ACGTTGCAAAAGTCCTCCAC	60.2	50.0	
C2E21_8175	Primer_8175_F	TTGTGCTGCAGTCCTTTGTC	60.0	50.0	180
	Primer_8175_R	CCTCCTCAAGCATGCTCTTC	60.1	55.0	
C2E21_0947	Primer_0947_F	ATCTGGGTGTACGGCAACTC	60.0	55.0	218
	Primer_0947_R	ACGTAAATGGCCTTCACCTG	60.0	50.0	
C2E21_4446	Primer_4446_F	TGGACATGCAGAAGAAGCTG	60.1	50.0	194
	Primer_4446_R	CTCAACAGGCTCTGGCTTG	59.7	57.9	
C2E21_8390	Primer_8390_F	CAGCCAGCAGCTGTCAGAG	61.1	63.2	184
	Primer_8390_R	CATGTATGAATCCGCCTTGA	59.5	45.0	
C2E21_9040	Primer_9040_F	GGAGCGCTACAACGAGTTTC	60.0	55.0	204
	Primer_9040_R	ACCACGTACGCATCCTTCTC	60.1	55.0	
C2E21_6023	Primer_6023_F	ACAATGTGCTGGTGGTTCAC	59.4	50.0	179
	Primer_6023_R	CAGAGAGAACAGCGATGCAG	59.9	55.0	
C2E21_2737	Primer_2737_F	ACGGTGTTGGTCAACTTTGG	60.8	50.0	161
	Primer_2737_R	AAGGTGGGGGTGTAGGTGAT	60.5	55.0	
C2E21_6815	Primer_6815_F	CGGCAGAGGAGTTCAAGAAG	60.1	55.0	184
	Primer_6815_R	TTCTCCACAAAGGGCTTGAG	60.4	50.0	
C2E21_3730	Primer_3730_F	TCCACTACAGCGACGACAAG	60.0	55.0	152
	Primer_3730_R	GAAGTTCATGAAGCCGTCGT	60.3	50.0	
18S (KR904895)[a]	Primer_18S_F	CCTGCGGCTTAATTTGACTC	59.8	50.0	192
	Primer_18S_R	GCGAACCAACCGTGACTATT	60.0	50.0	

[a]GenBank accession number

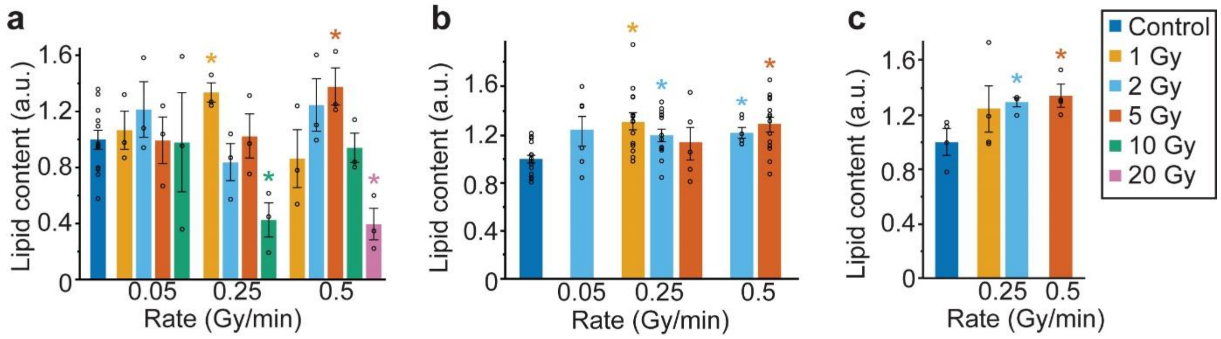

Supplementary Figure 1. Volumetric lipid content.
(**a**) Relative lipid content in the culture at 30 days after irradiation that was applied in the early exponential phase of culture growth. The number of biologically independent experiments was *n* = 12 for control and *n* = 3 for treatments. (**b**) Relative lipid content in the culture at 10 days after irradiation that was applied in the early stationary phase of culture growth. The number of biologically independent experiments was *n* = 16 for control and *n* = 5–14 for treatments. (**c**) Relative lipid content in the culture at 24 h after irradiation that was applied in the early stationary phase of culture growth. Relative lipid content was determined by Nile Red fluorescence (a.u., arbitrary units). The number of biologically independent experiments was *n* = 4. All data are means ± standard error. All data are means ± standard error. Significant difference compared to the control (non-irradiated) treatment was evaluated using a non-parametric two-tailed Mann–Whitney U test and is indicated at $p < 0.05$ (*).

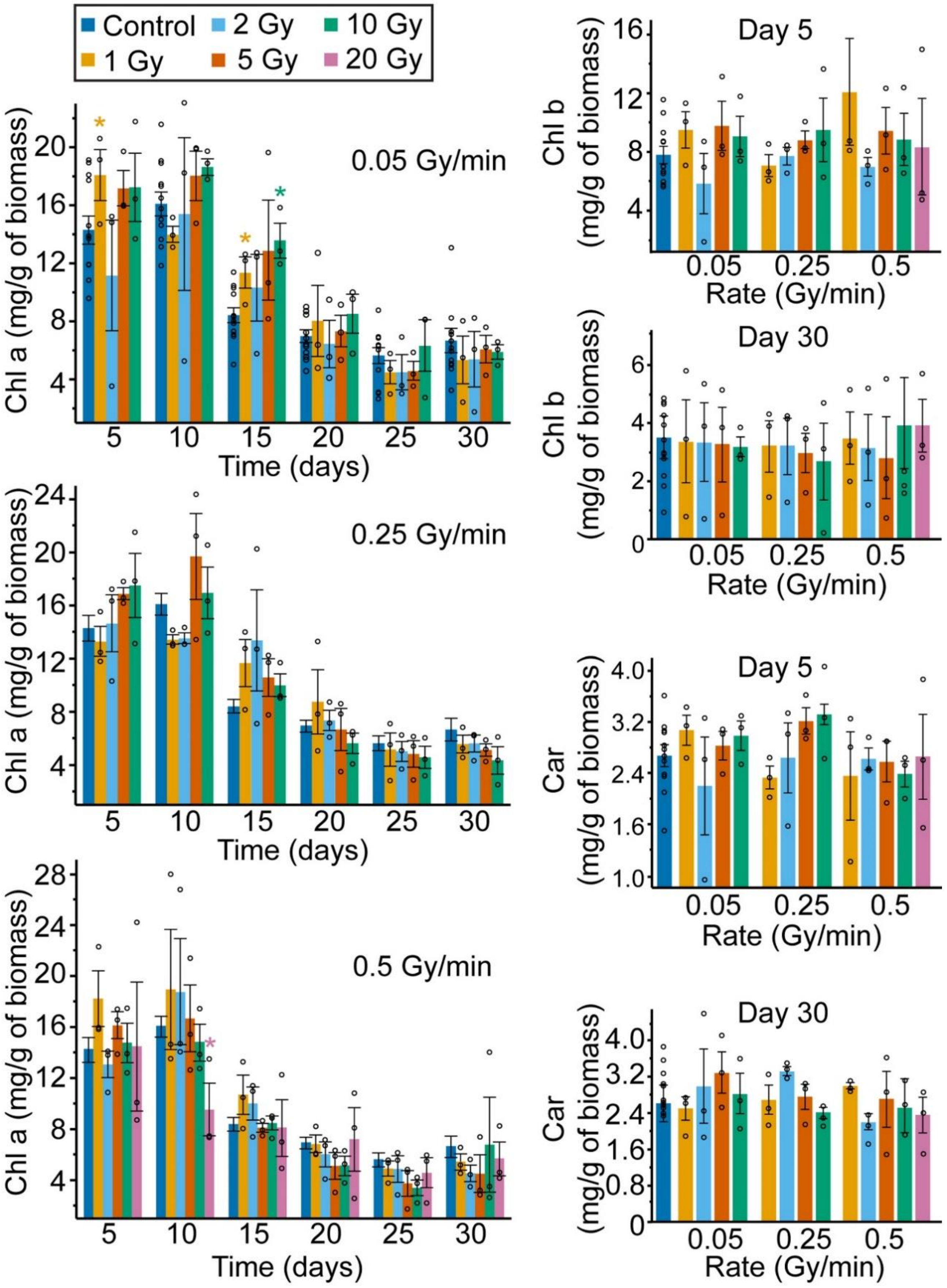

Supplementary Figure 2. Photosynthetic pigments analysis of cultures that were untreated or irradiated in the early exponential phase.

The analyses were performed at 5 – 30 days after irradiation. Chlorophyll-a, chl a; chlorophyll-b, chl b; carotenoids, car. The number of biologically independent experiments was $n = 12$ for controls and $n = 3$ for treatments. All data are means ± standard error. Significant difference compared to the control (non-irradiated) treatment was evaluated using a non-parametric two-tailed Mann–Whitney U test and is indicated at $p < 0.05$ (*).

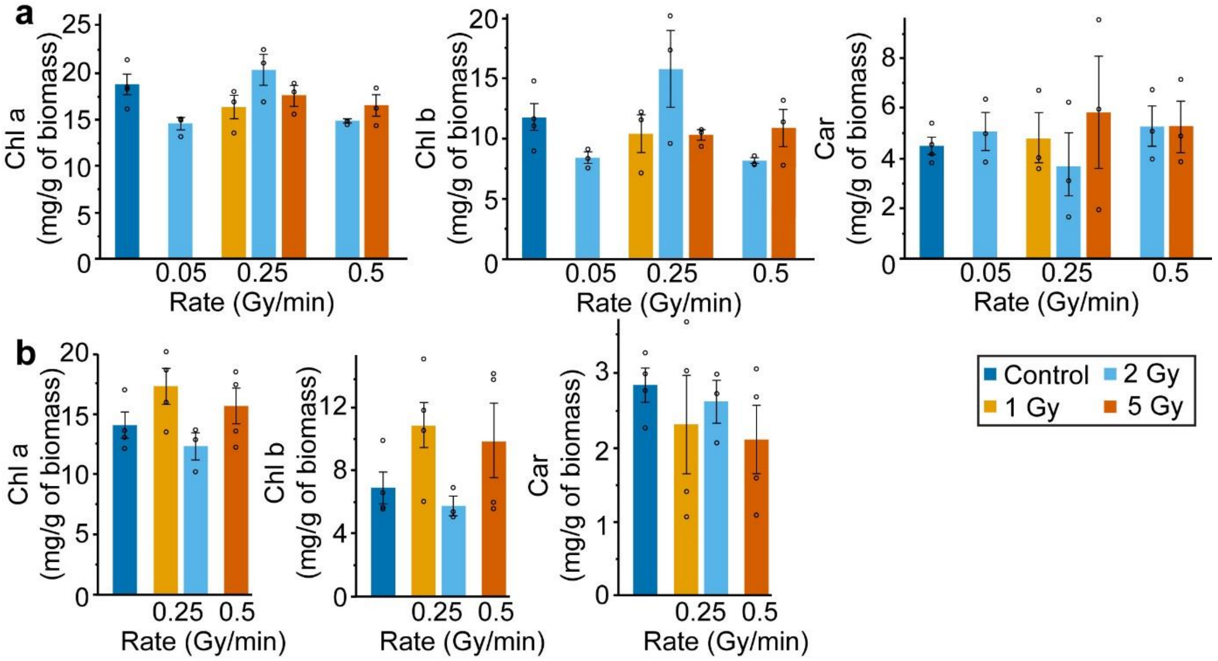

Supplementary Figure 3. Photosynthetic pigments analysis of cultures.
(**a**) Pigments 10 days after irradiation in the early stationary phase. (**b**) Pigments 1 day after irradiation in the early stationary phase. Irradiation was applied at day 20 after inoculation. Chlorophyll-a, chl a; chlorophyll-b, chl b; carotenoids, car. All data are means ± standard error. The number of biologically independent experiments was n = 4 for control and n = 3 for treatments. No statistical significance compared to the control (non-irradiated) treatment was observed, evaluated using a non-parametric two-tailed Mann–Whitney U test.

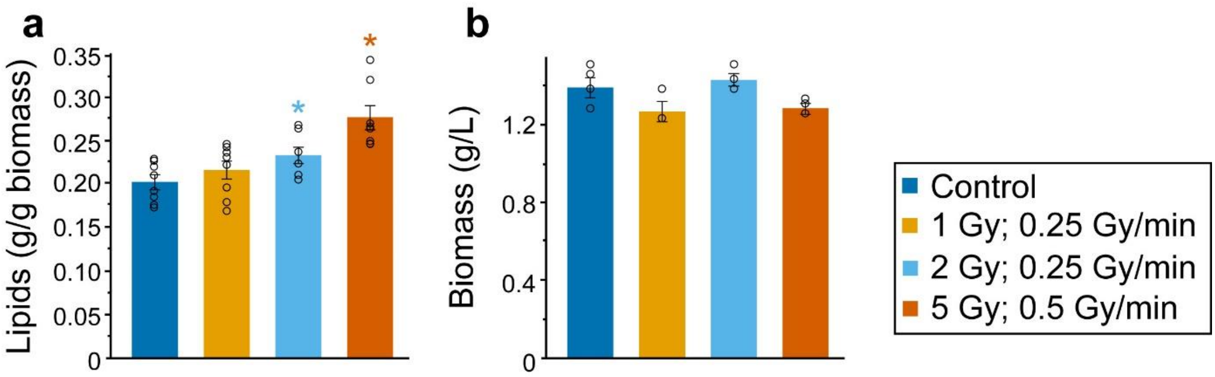

Supplementary Figure 4. Effects of irradiation applied in early stationary phase measured 10 days later.

(**a**) Lipid content in biomass determined by lipid extraction and gravimetry. The number of biologically independent experiments was $n = 8$. (**b**) Biomass. The number of biologically independent experiments was $n = 4$ for control, and $n = 3$ for treatments. All data are means ± standard error. Significant difference compared to the control (non-irradiated) treatment was evaluated using a non-parametric two-tailed Mann–Whitney U test and is indicated at $p < 0.05$ (*).

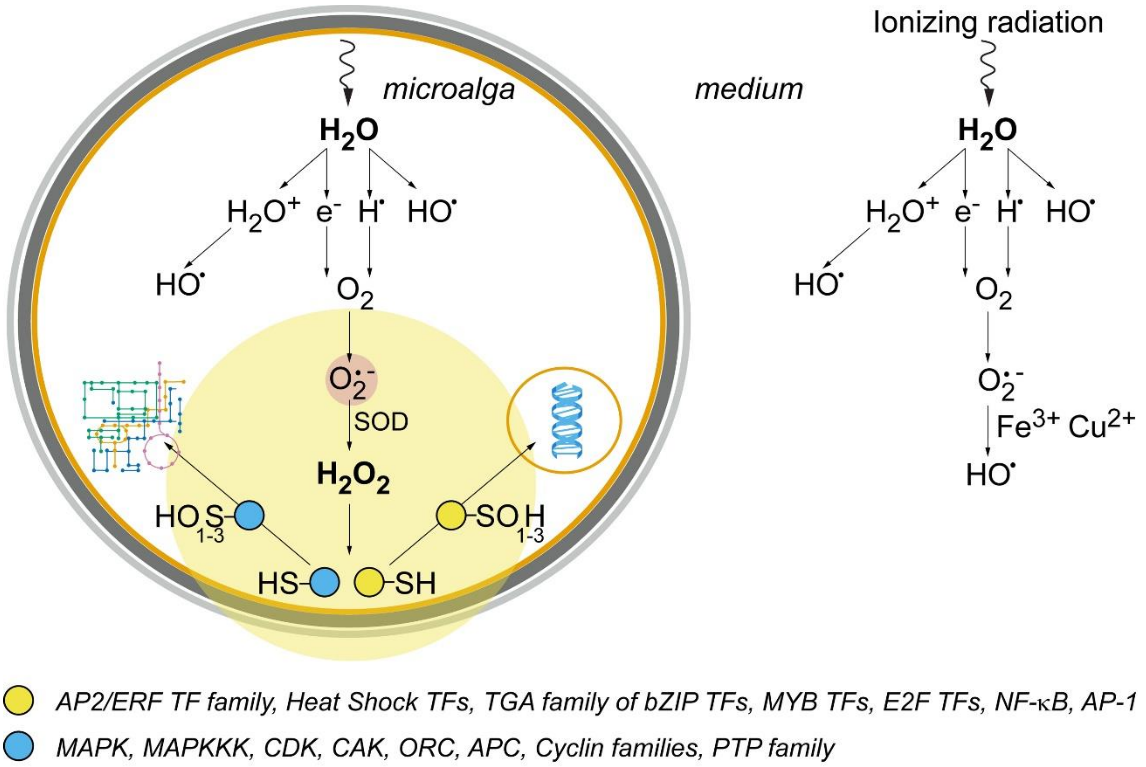

Supplementary Figure 5. The main redox routes induced by ionizing radiation in aqueous oxygenated environment at neutral pH.

This simplified scheme was made according to known reaction rates and reactants concentrations and yields (Joseph, et al., 2008 *Radiat. Phys. Chem*. 77, 1009–1020). Colored circles around hydrogen peroxide (H_2O_2) and superoxide radical anion ($O_2^{•-}$) illustrate their diffusion radii and capacities to reach targets in other compartments. For hydroxyl radical ($HO^•$), it is only ~3 nm (as compared to H_2O_2 with radius >1 µm). $HO^•$ is very short-lived and induces damage near the site of production. It cannot enter the cell from the medium and it is mainly scavenged by cell wall and mucilage. In the medium, $O_2^{•-}$ reacts with free/loosely bound metals to give rise to $HO^•$ and other products. In the cell, redox-active metals are sequestrated/inactivated, so the main fate of $O_2^{•-}$ is dismutation by superoxide dismutases (SOD) to H_2O_2. H_2O_2 shows half-life time of ~5 ms, and can pass membranes. It is a pleiotropic signalling molecule that controls a set of transcription factors (TFs; yellow circles) and the activity of proteins in different metabolic pathways (blue circles), through multi-step oxidative modifications of thiol switches (-SH to -SOH, -SO_2H, or -SO_3H). TFs and proteins are listed that appear to be redox regulated in plants and are present in *Chlorella*.

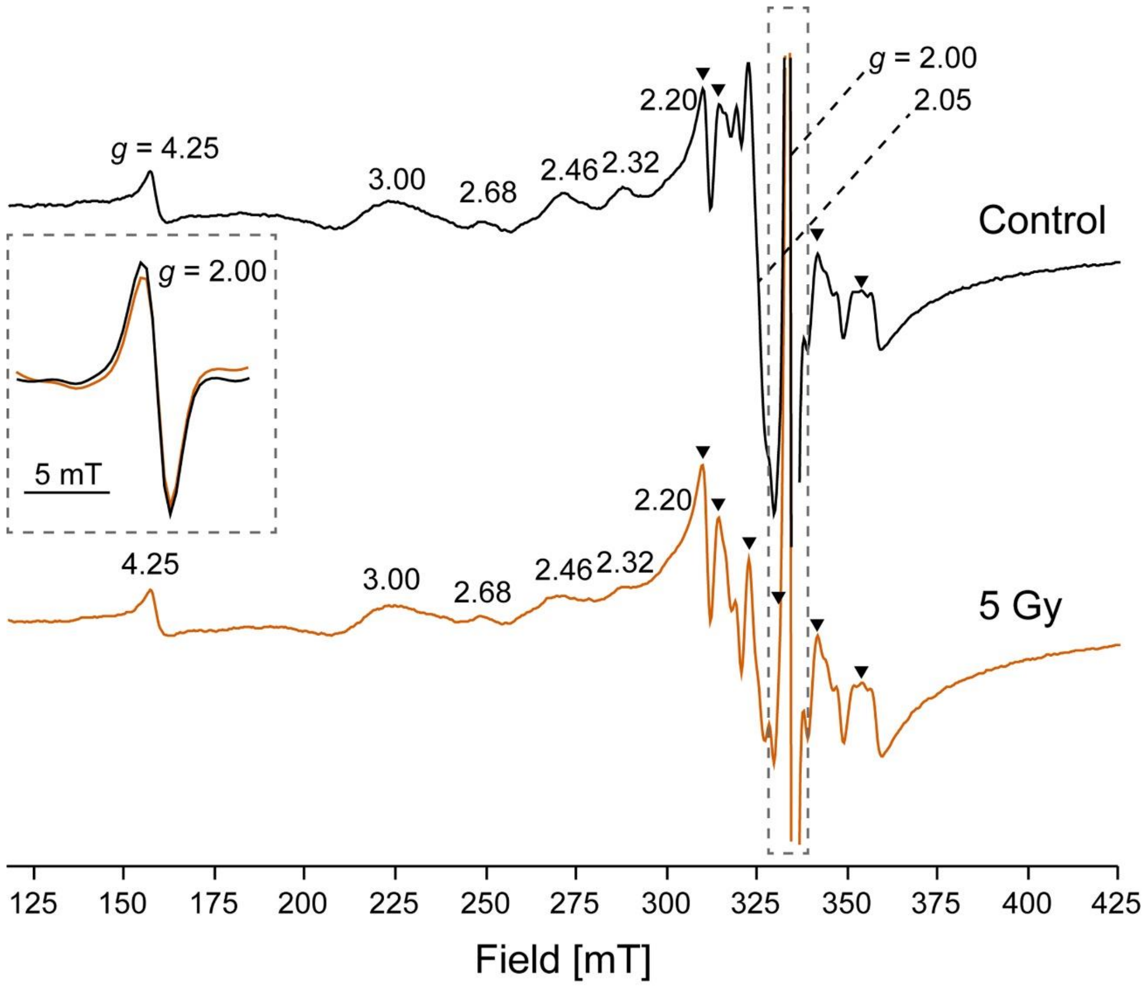

Supplementary Figure 6. 19 K EPR spectra of *C. sorokiniana* fresh biomass before and after irradiation with 5 Gy.

g-Values are assigned as follows: 4.25 – non-specifically bound high-spin Fe^{3+}; 3.00 and 2.46 – low-spin Fe^{3+} in heme; 2.68, 2.46, 2.32 and 2.20 – four lines coming from hyperfine coupling of Cu^{2+} along parallel axis; 2.05 – *g*-perpendicular line of for Cu^{2+}. 2.00 - organic radical (enlargement is presented in the box); triangles - characteristic 6-line EPR signal of Mn^{2+}. Irradiation resulted in the reduction of Cu^{2+} signal to 'EPR silent' Cu^{1+}.

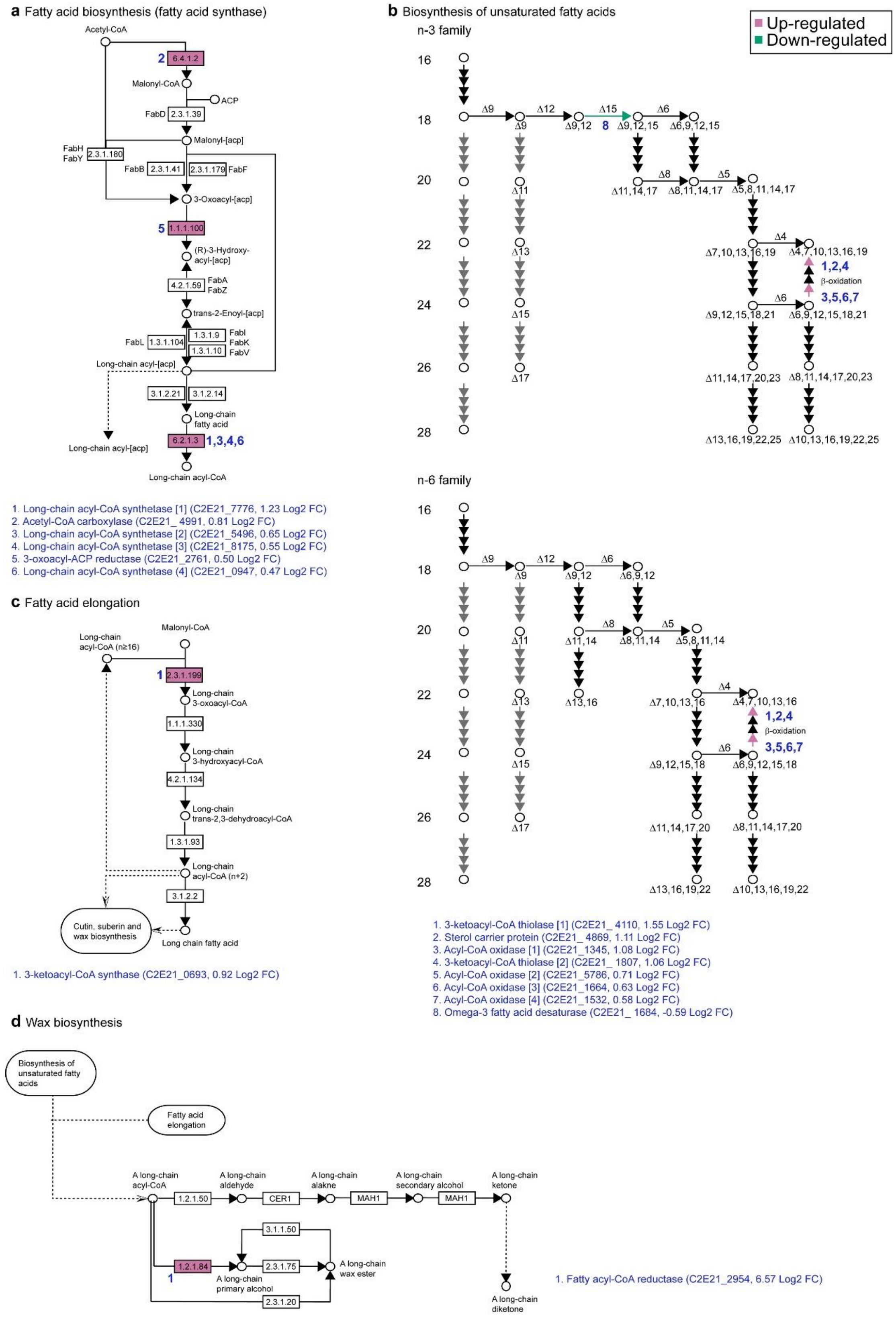

Supplementary Figure 7. Mapping of lipid metabolism enzymes that were significantly up- and down-regulated in response to irradiation treatment.

Mapping using KEGG pathway analysis. Log2 fold-change (FC) values of each transcript are indicated. Many up-regulated transcripts encode components of the chloroplastic fatty acid synthase pathway (**a**), the long-chain unsaturated fatty acids biosynthesis pathway (**b**), fatty acid elongation (**c**) and the wax biosynthesis pathway (**d**) (continued on next page).

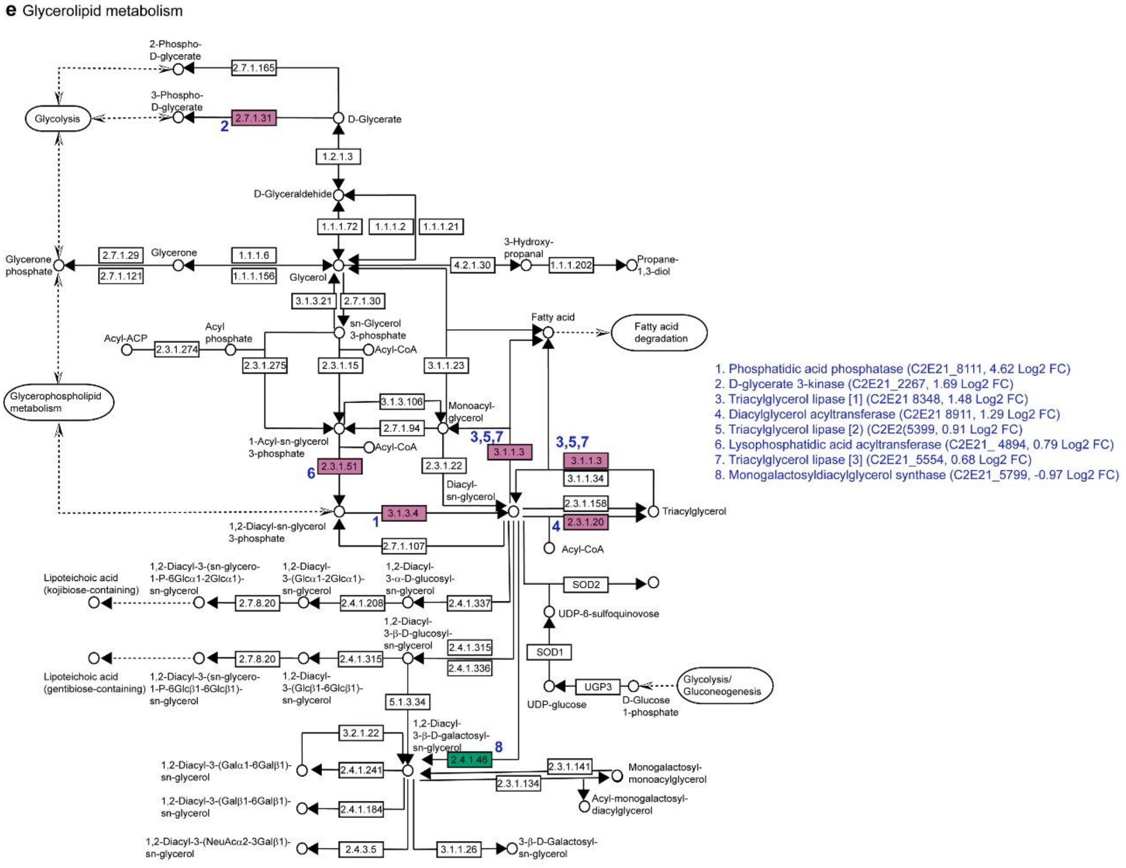

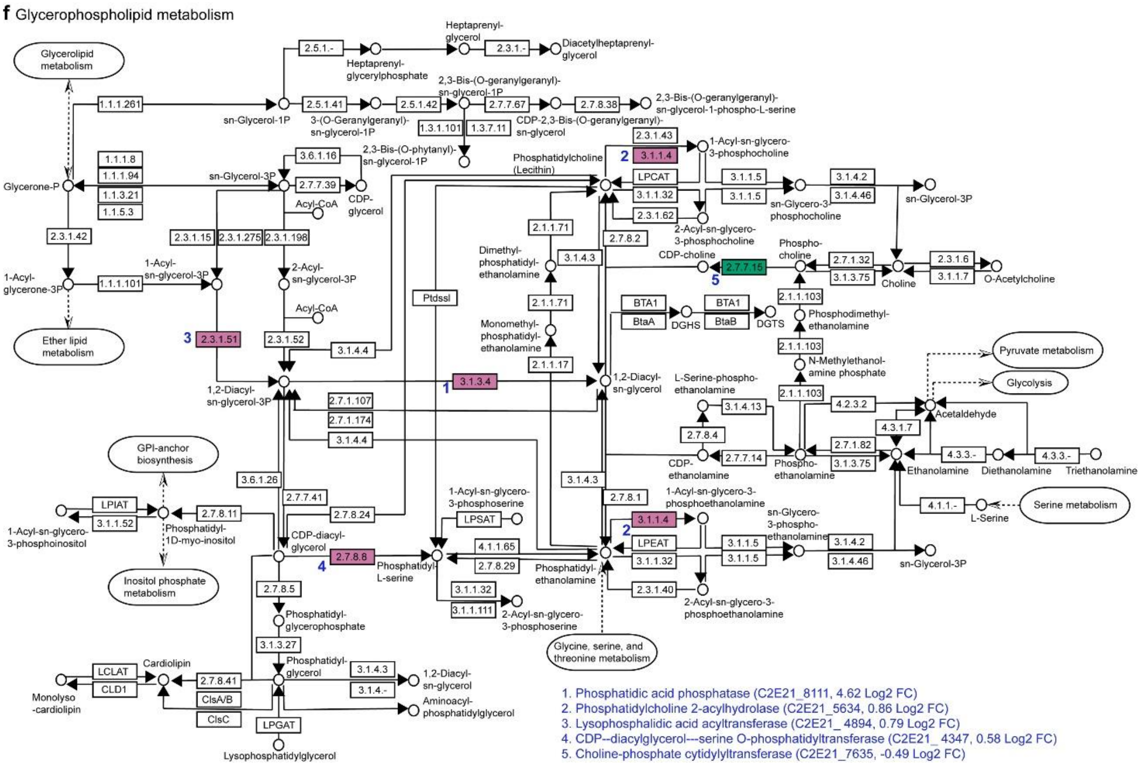

Supplementary Figure 7 (continued). Up-regulated transcripts that encode key enzymes for triacylglycerol (TAG) were mapped in glycerolipid biosynthesis pathway (**e**) and glycerophospholipid pathway (**f**) (continued on next page).

g Fatty acid degradation

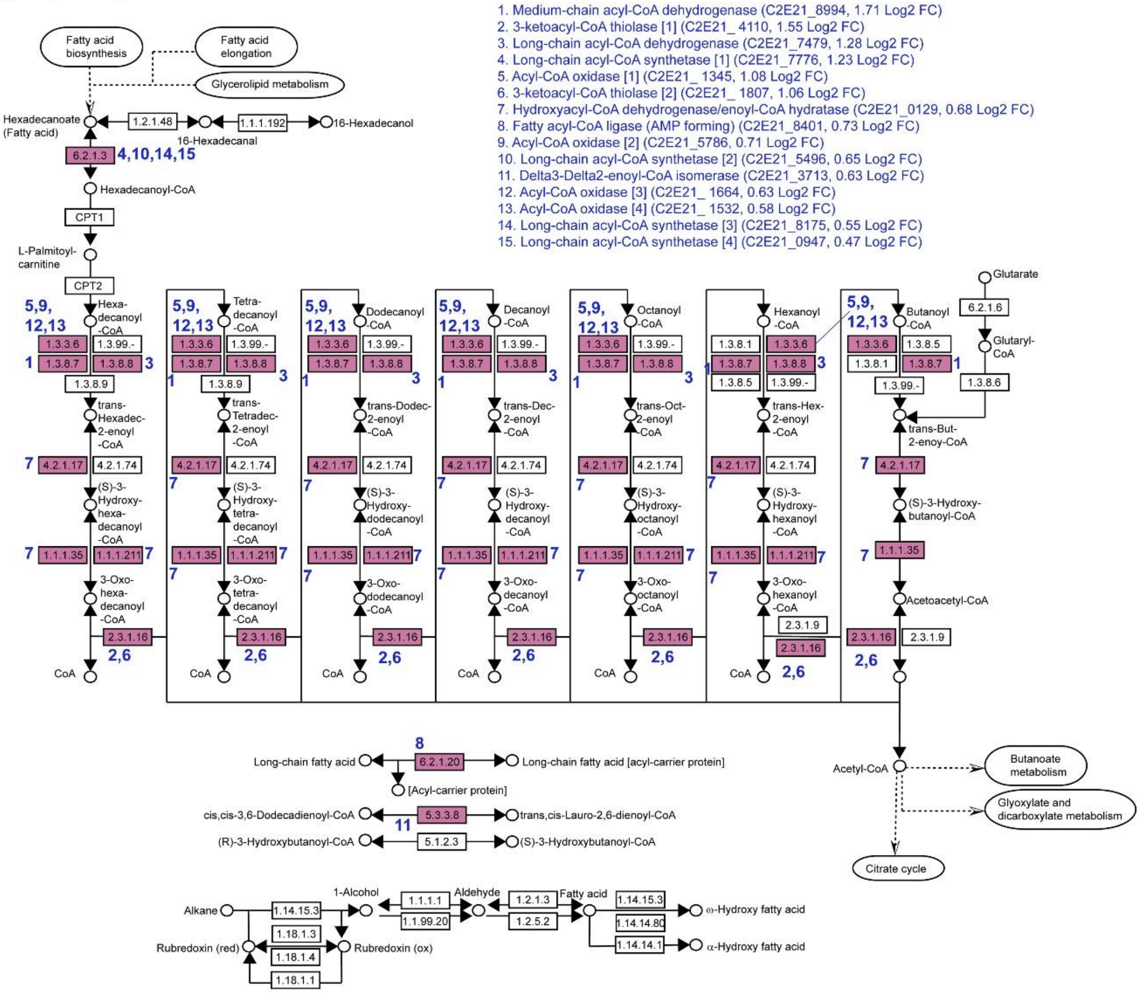

1. Medium-chain acyl-CoA dehydrogenase (C2E21_8994, 1.71 Log2 FC)
2. 3-ketoacyl-CoA thiolase [1] (C2E21_ 4110, 1.55 Log2 FC)
3. Long-chain acyl-CoA dehydrogenase (C2E21_7479, 1.28 Log2 FC)
4. Long-chain acyl-CoA synthetase [1] (C2E21_7776, 1.23 Log2 FC)
5. Acyl-CoA oxidase [1] (C2E21_ 1345, 1.08 Log2 FC)
6. 3-ketoacyl-CoA thiolase [2] (C2E21_ 1807, 1.06 Log2 FC)
7. Hydroxyacyl-CoA dehydrogenase/enoyl-CoA hydratase (C2E21_0129, 0.68 Log2 FC)
8. Fatty acyl-CoA ligase (AMP forming) (C2E21_8401, 0.73 Log2 FC)
9. Acyl-CoA oxidase [2] (C2E21_5786, 0.71 Log2 FC)
10. Long-chain acyl-CoA synthetase [2] (C2E21_5496, 0.65 Log2 FC)
11. Delta3-Delta2-enoyl-CoA isomerase (C2E21_3713, 0.63 Log2 FC)
12. Acyl-CoA oxidase [3] (C2E21_ 1664, 0.63 Log2 FC)
13. Acyl-CoA oxidase [4] (C2E21_ 1532, 0.58 Log2 FC)
14. Long-chain acyl-CoA synthetase [3] (C2E21_8175, 0.55 Log2 FC)
15. Long-chain acyl-CoA synthetase [4] (C2E21_0947, 0.47 Log2 FC)

h Sphingolipid metabolism

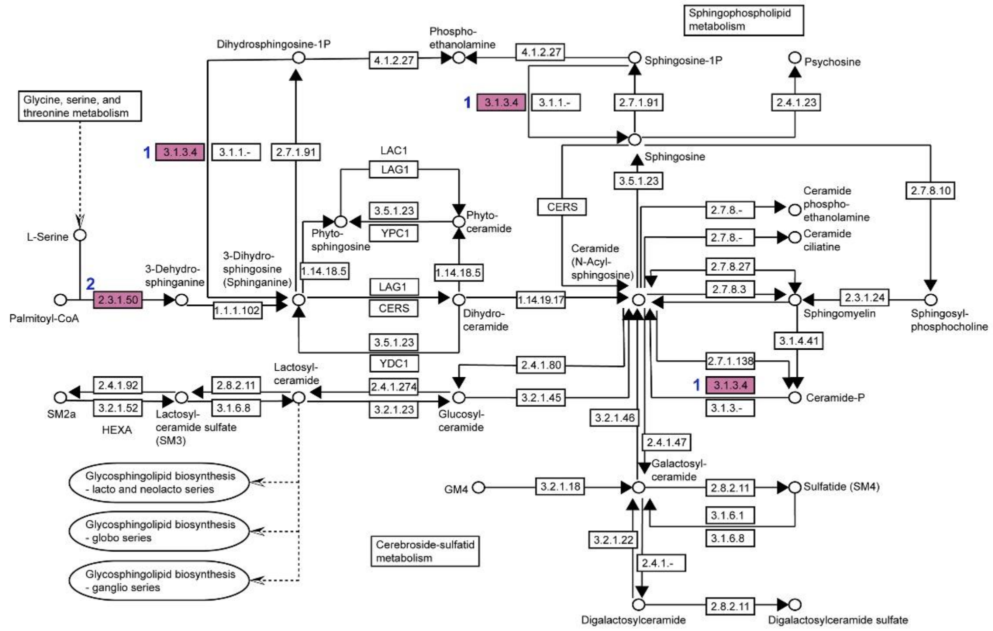

1. Phosphatidic acid phosphatase (C2E21_8111, 4.62 Log2 FC)
2. Serine palmitoyltransferase (C2E21_ 6382, 1.11 Log2 FC)

Supplementary Figure 7 (continued). Many up-regulated transcripts were also mapped to the fatty acid degradation pathway (**g**) and the sphingolipid metabolism pathway (**h**).

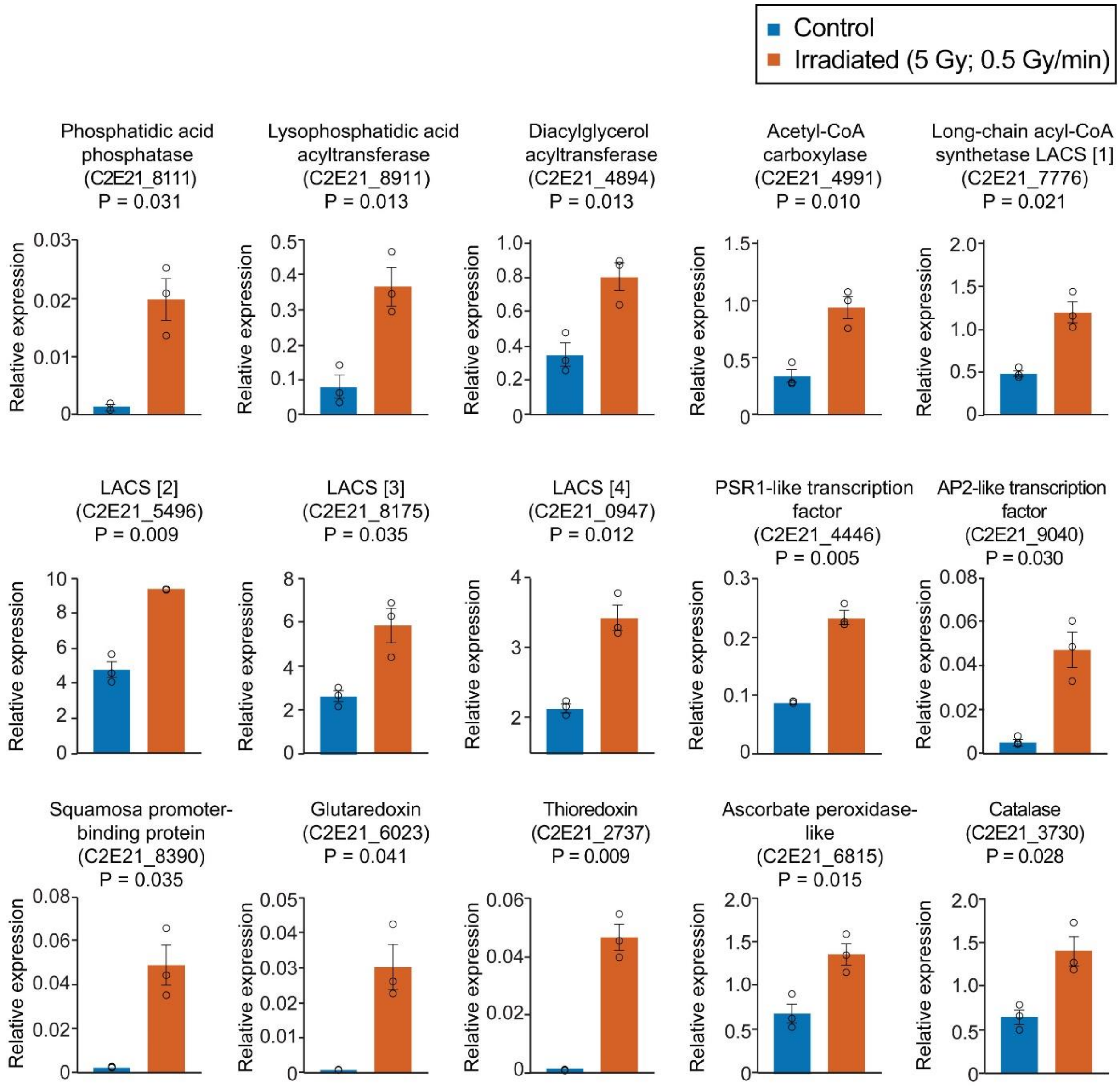

Supplementary Figure 8. Relative expression by qPCR of selected genes related to lipid metabolism, transcriptional regulation and reactive oxygen species responses following irradiation.

Expression levels were determined relative to *18S* expression. All data are means ± standard error and the number of biologically independent experiments was $n = 3$. The differences compared to control are all significant at $p < 0.05$ as evaluated using non-parametric two-tailed Mann–Whitney U tests, and as indicated on each graph panel.

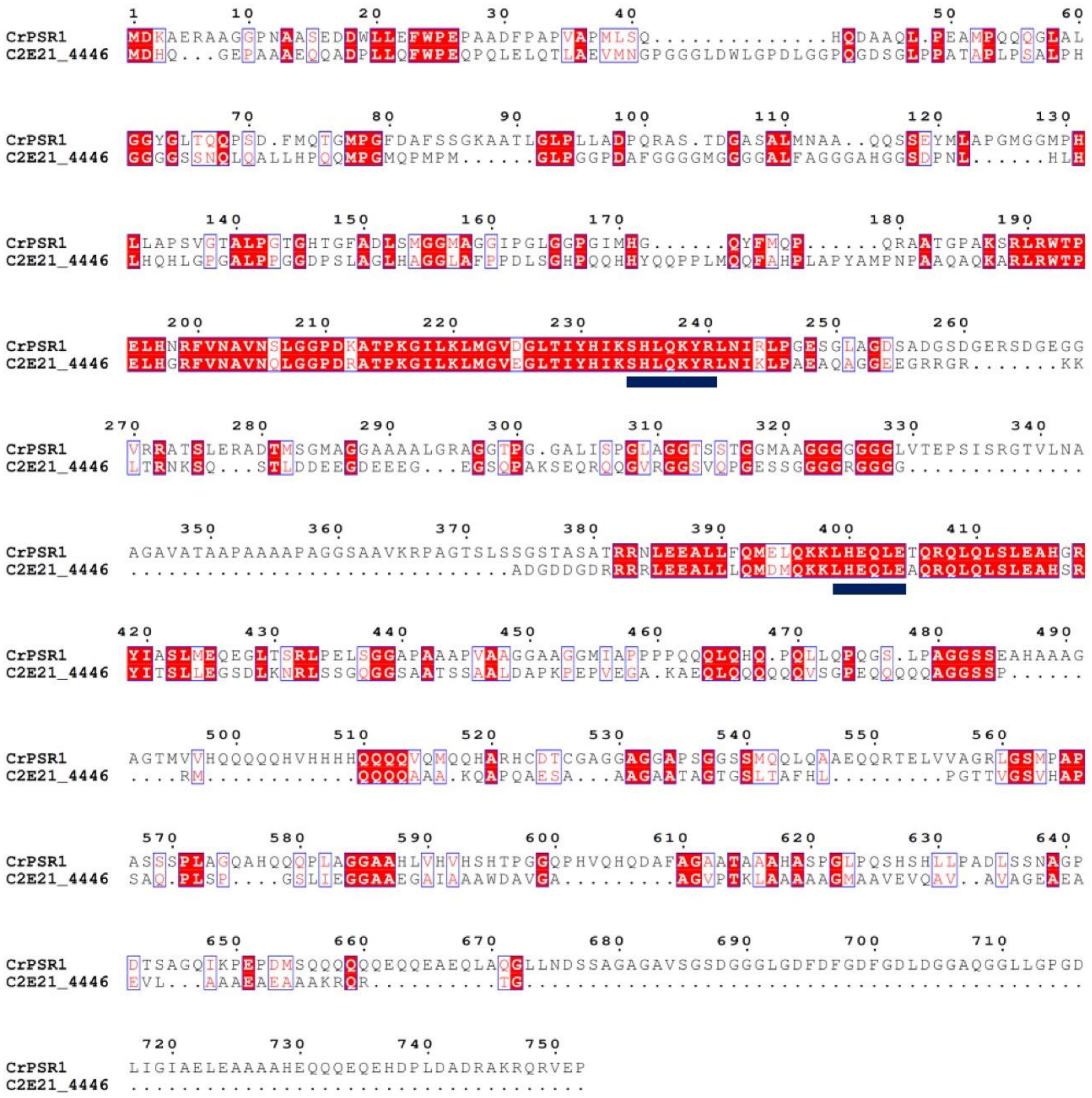

Supplementary Figure 9. The comparison of amino acid sequences of *C. sorokiniana* PSR1 ortholog (C2E21_4446) and *C. reinhardtii* PSR1 (CrPSR1).
Pairwise amino acid sequence alignment shows that C2E21_4446 has high similarity with CrPSR1 and the conserved SH[A/L]QKY[R/F] motif within MYB-like DNA-binding domain, and the conserved LHEQLE motif within the coiled-coil protein dimerization transfactor domain (both highlighted) are both present. Amino acids that are identical or similar are in red shading or red font, respectively.

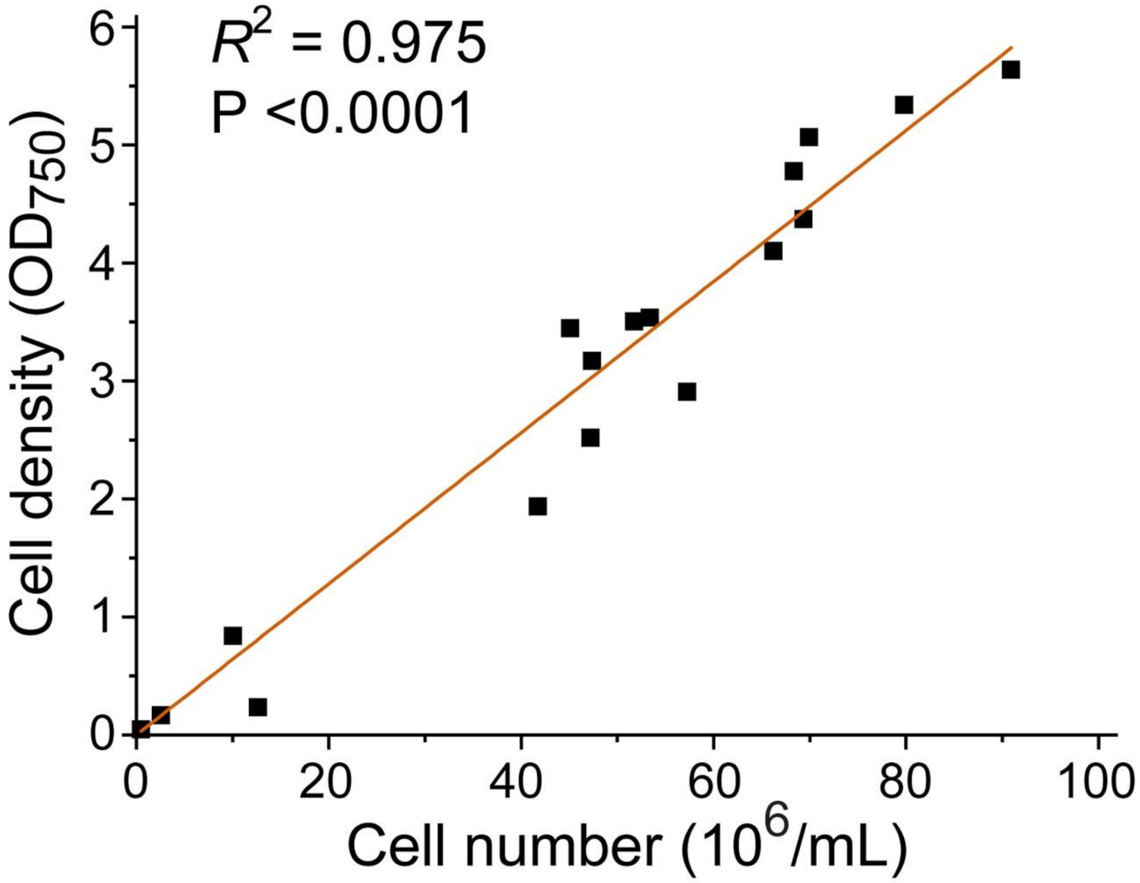

Supplementary Figure 10. Linear correlation between optical density at 750 nm (OD_{750}), and cell number of *C. sorokiniana* cultures during 30 days of growth.
Pearson's correlation coefficient (R^2) was used to determine the correlation between both parameters. All data are mean values (n = 3).

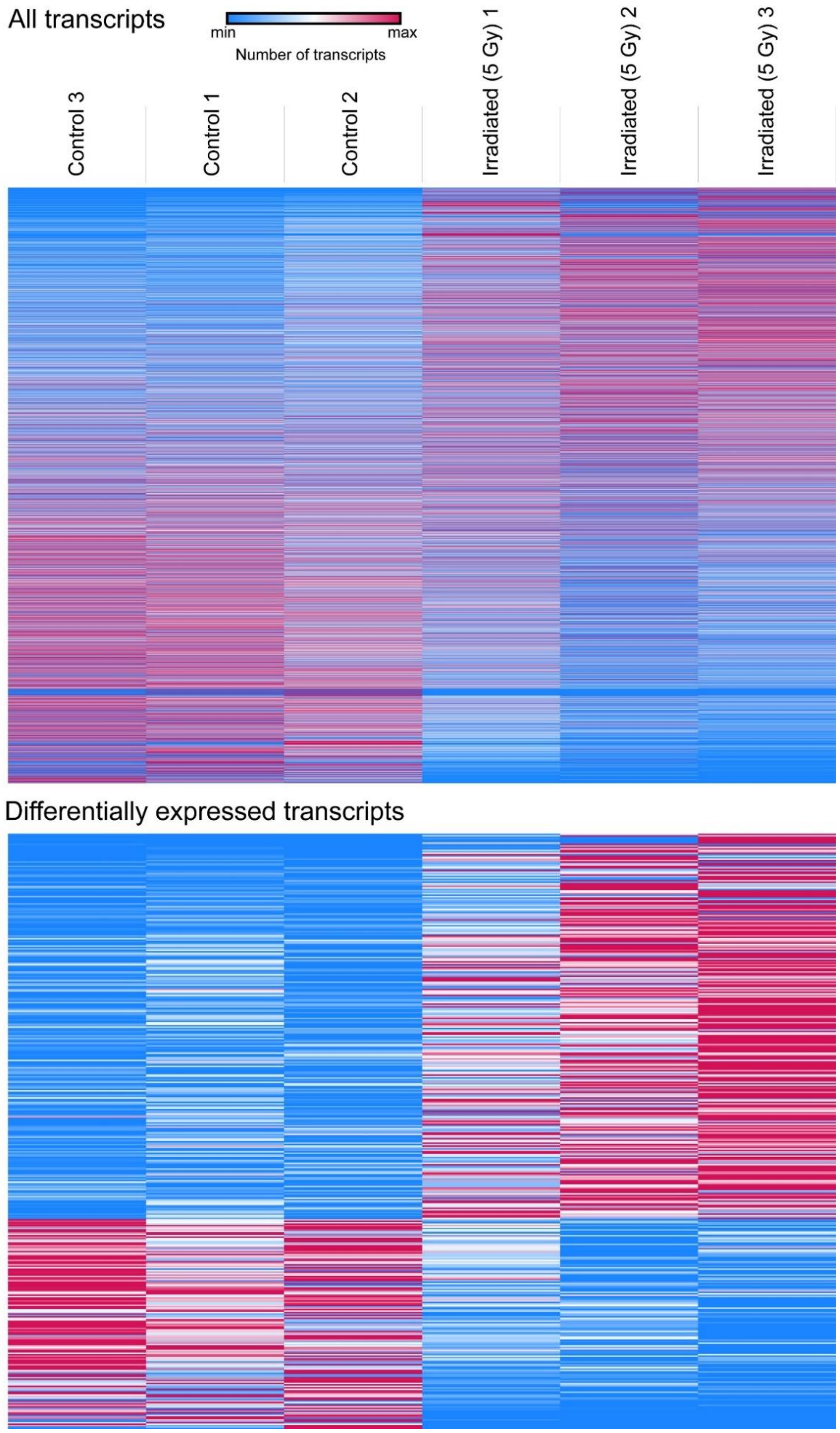

Supplementary Figure 11. Heatmaps of control and irradiation treated transcripts. Heatmap analysis of all transcripts (*n* = 9526) and all significantly differentially expressed transcripts (FDR < 0.05; *n* = 410) from *C. sorokiniana* that was untreated (control) or exposed to ionizing radiation (dose 5 Gy; dose rate 0.5 Gy/min).
