# Supplementary material for: Low-dose ionizing radiation generates a hormetic response to modify lipid metabolism in Chlorella sorokiniana
Source: Commun Biol. 2024 Jul 6;7:821. doi: 10.1038/s42003-024-06526-6 (PMC11226653; doi:10.1038/s42003-024-06526-6)
Supplement: Supplementary file 3 — Description of Additional Supplementary Files [file 42003_2024_6526_MOESM3_ESM.pdf]

Description of Additional Supplementary Files

File name: Supplementary Data 1

Description: Expression data of significantly differentially expressed transcripts

File name: Supplementary Data 2

Description: The source data behind the graphs
