# Supplementary material for: Low-dose ionizing radiation generates a hormetic response to modify lipid metabolism in Chlorella sorokiniana
Source: Commun Biol. 2024 Jul 6;7:821. doi: 10.1038/s42003-024-06526-6 (PMC11226653; doi:10.1038/s42003-024-06526-6)
Supplement: Supplementary file 6 — Reporting Summary [file 42003_2024_6526_MOESM6_ESM.pdf]

nature portfolio

Corresponding author(s): Ivan Spasojević and Jon K. Pittman

Last updated by author(s): May 14, 2024

Reporting Summary

Nature Portfolio wishes to improve the reproducibility of the work that we publish. This form provides structure for consistency and transparency in reporting. For further information on Nature Portfolio policies, see our Editorial Policies and the Editorial Policy Checklist.

Statistics

For all statistical analyses, confirm that the following items are present in the figure legend, table legend, main text, or Methods section.

n/a	Confirmed	
☐	☒	The exact sample size (*n*) for each experimental group/condition, given as a discrete number and unit of measurement
☐	☒	A statement on whether measurements were taken from distinct samples or whether the same sample was measured repeatedly
☐	☒	The statistical test(s) used AND whether they are one- or two-sided<br>*Only common tests should be described solely by name; describe more complex techniques in the Methods section.*
☒	☐	A description of all covariates tested
☒	☐	A description of any assumptions or corrections, such as tests of normality and adjustment for multiple comparisons
☐	☒	A full description of the statistical parameters including central tendency (e.g. means) or other basic estimates (e.g. regression coefficient) AND variation (e.g. standard deviation) or associated estimates of uncertainty (e.g. confidence intervals)
☐	☒	For null hypothesis testing, the test statistic (e.g. *F*, *t*, *r*) with confidence intervals, effect sizes, degrees of freedom and *P* value noted<br>*Give P values as exact values whenever suitable.*
☒	☐	For Bayesian analysis, information on the choice of priors and Markov chain Monte Carlo settings
☒	☐	For hierarchical and complex designs, identification of the appropriate level for tests and full reporting of outcomes
☐	☒	Estimates of effect sizes (e.g. Cohen's *d*, Pearson's *r*), indicating how they were calculated

Our web collection on statistics for biologists contains articles on many of the points above.

Software and code

Policy information about availability of computer code

Data collection	Fluorescence spectra were collected using FluorEssence 3.5 software (Horiba). EPR spectra were collected using XEPR (Bruker). GC chromatograms were collected using GCMSsolution Ver. 2 software (Shimatzu). TEM micrographs were collected using AMT Image Capture Engine (AMT). RNA-Seq transcriptomics data were obtained by sequence data demultiplexing and BCL-to-Fastq conversion using Illumina bcl2fastq software, version 2.20.0.422, after sequencing on the Illumina HiSeq4000 platform. Real-time PCR data were collected using StepOne™ software v2.3 (Thermo Fisher Scientific).
Data analysis	The following publically available and/or published software were used for data analysis as described in the Methods: ImageJ 1.53, FluorEssence 3.5, XEPR, GCMSsolution V.2, Trimmomatic 0.40, STAR mapper, HTSeq, Python 3.7, DESeq2, Morpheus, BLAST, KEGG Mapper, Clustal Omega, ESPript 3.0, STATISTICA 8.0. No custom code was used for the analysis.

For manuscripts utilizing custom algorithms or software that are central to the research but not yet described in published literature, software must be made available to editors and reviewers. We strongly encourage code deposition in a community repository (e.g. GitHub). See the Nature Portfolio guidelines for submitting code & software for further information.

Data

Policy information about availability of data

All manuscripts must include a data availability statement. This statement should provide the following information, where applicable:

- Accession codes, unique identifiers, or web links for publicly available datasets
- A description of any restrictions on data availability
- For clinical datasets or third party data, please ensure that the statement adheres to our policy

Sequence data from this article can be found in the EMBL-EBI ArrayExpress data library under accession number E-MTAB-12288. All raw source data are available in Supplementary Data 1 and Supplementary Data 2 (Source Data) files.

Human research participants

Policy information about studies involving human research participants and Sex and Gender in Research.

Reporting on sex and gender	*Use the terms sex (biological attribute) and gender (shaped by social and cultural circumstances) carefully in order to avoid confusing both terms. Indicate if findings apply to only one sex or gender; describe whether sex and gender were considered in study design whether sex and/or gender was determined based on self-reporting or assigned and methods used. Provide in the source data disaggregated sex and gender data where this information has been collected, and consent has been obtained for sharing of individual-level data; provide overall numbers in this Reporting Summary. Please state if this information has not been collected. Report sex- and gender-based analyses where performed, justify reasons for lack of sex- and gender-based analysis.*
Population characteristics	*Describe the covariate-relevant population characteristics of the human research participants (e.g. age, genotypic information, past and current diagnosis and treatment categories). If you filled out the behavioural & social sciences study design questions and have nothing to add here, write "See above."*
Recruitment	*Describe how participants were recruited. Outline any potential self-selection bias or other biases that may be present and how these are likely to impact results.*
Ethics oversight	*Identify the organization(s) that approved the study protocol.*

Note that full information on the approval of the study protocol must also be provided in the manuscript.

Field-specific reporting

Please select the one below that is the best fit for your research. If you are not sure, read the appropriate sections before making your selection.

☒ Life sciences ☐ Behavioural & social sciences ☐ Ecological, evolutionary & environmental sciences

For a reference copy of the document with all sections, see nature.com/documents/nr-reporting-summary-flat.pdf

Life sciences study design

All studies must disclose on these points even when the disclosure is negative.

Sample size	Sample sizes were determined based on previous experience with similar successful experiments, and in all cases at least three independent replicate cultures, each containing a large population of cells were used for analysis. The sample size was considered to be sufficient because the methods used a highly reproducible. No statistical tests were used to determine sample size.
Data exclusions	No data were excluded from the study.
Replication	All attempts at replication were successful.
Randomization	Randomization was mostly not relevant to the experimental design of the study as identical cell cultures were used throughout. There was randomization of placement position of culture flasks from different treatment groups placed in the growth cabinet to ensure that any variation in environmental conditions was equivalent between treatments and independent replicates.
Blinding	Blinding was not performed in this study because the quantification methods used in this study such as RNA-Seq or GC-MS are not influenced by human interpretation or bias.

Reporting for specific materials, systems and methods

We require information from authors about some types of materials, experimental systems and methods used in many studies. Here, indicate whether each material, system or method listed is relevant to your study. If you are not sure if a list item applies to your research, read the appropriate section before selecting a response.

Materials & experimental systems

n/a	Involved in the study
☒	☐ Antibodies
☐	☒ Eukaryotic cell lines
☒	☐ Palaeontology and archaeology
☒	☐ Animals and other organisms
☒	☐ Clinical data
☒	☐ Dual use research of concern

Methods

n/a	Involved in the study
☒	☐ ChIP-seq
☒	☐ Flow cytometry
☒	☐ MRI-based neuroimaging

Eukaryotic cell lines

Policy information about cell lines and Sex and Gender in Research

Cell line source(s)	Chlorella sorokiniana strain CCAP 211/8K algae cells were obtained from the Culture Collection of Algae and Protozoa (CCAP), Oban, UK.
Authentication	Cells were obtained from a trusted national culture collection (CCAP) and were authenticated by transcriptome sequencing.
Mycoplasma contamination	Only algae were used in this study and no animal components therefore cells were not tested for mycoplasma contamination.
Commonly misidentified lines (See ICLAC register)	No commonly misidentified cell lines were used in the study.
